# Supplementary material for: CLEAR: coverage-based limiting-cell experiment analysis for RNA-seq
Source: J Transl Med. 2020 Feb 10;18:63. doi: 10.1186/s12967-020-02247-6 (PMC7008572; doi:10.1186/s12967-020-02247-6)
Supplement: Supplementary file 1 — Additional file 1: Figure S1. FACS parameter diagrams for the enrichment of CD5+ and CD5− cells from a CLL patient PBMC sample. FACS flow diagrams depicting the steps involved in the enrichment of CD5+ cells and CD5− cells from live CD45+ , CD3−, CD33−, CD19+ cells, followed by separation into either CD5+ or CD5− collection tubes (Fig. 1). FACS: Fluorescence Activated Cell Sorting. Figure S2. Survey of quality control (QC) metrics of RNA-Seq data. All sequencing was subject to quality control as described in “Methods”. Key metrics are summarized here. Notably, in many metrics such as Intergenic Rate, Alignment Rate, and Duplication Rate, the 10-pg groups indicate lower quality libraries than 100-pg and 1000-pg. “Top30” corresponds to the proportion of reads that belong to the 30 highest genes by expression. Boxplots: orange line, mean metric value; whiskers: displaying 1.5× the inter-quartile range (IQR) beyond the first and the third quartiles; circles: outliers. Figure S3. Between-sample correlations of detected RNA-Seq read counts. Scatter plots are drawn comparing each sample to each other sample for each input mass. 10-pg samples show much more scattered counts, whereas 100-pg and 1000-pg samples show progressively higher correlation. Figure S4. Comparison of overlapping transcripts. The analysis from Fig. 3a was repeated, although CD5− and CD5+ samples were considered separately. Notably, the trend between CD5+ and CD5− mirrors that of the pooled data in Fig. 3a. Figure S5. CLEAR Filtering results in fewer noisy transcripts at the 10-pg sample level. Analysis from Figure S3 was repeated using CLEAR-filtered gene counts. Notably, 10-pg samples are observed to be sparser, while the remaining data points are of much higher correlation. Figure S6. Application of CLEAR to public datasets. A, B data from Ilicic et al. [25] was processed using the CLEAR pipeline; C, D data from Bhargava et al. [14] was processed using the CLEAR pipeline; A) An example CLEAR trace fro [file 12967_2020_2247_MOESM1_ESM.pdf]

Supplementary Material For:

CLEAR: Coverage-based Limiting-cell Experiment Analysis for RNA-seq

Logan A Walker[1,2], Michael G Sovic[2], Chi-Ling Chiang[2,3], Eileen Hu[2,3], Jiyeon K Denninger[4], Xi Chen[2], Elizabeth D Kirby[4,5], John C Byrd[2,3], Natarajan Muthusamy[2,3], Ralf Bundschuh[1,3,6,7]*, & Pearlly Yan[2,3]*

[1] *Department of Physics, College of Arts and Sciences*

[2] *The Ohio State University Comprehensive Cancer Center*

[3] *Division of Hematology, Department of Internal Medicine, College of Medicine*

[4] *Department of Psychology, College of Arts and Sciences*

[5] *Chronic Brain Injury Program, The Ohio State University*

[6] *Department of Chemistry & Biochemistry, College of Arts and Sciences*

[7] *Center for RNA Biology, The Ohio State University, Columbus, OH*

* To whom correspondence should be addressed. Tel: +1 614 688 3978 and +1 614 685 9164; Email: bundschuh@mps.ohio-state.edu and Pearlly.Yan@osumc.edu

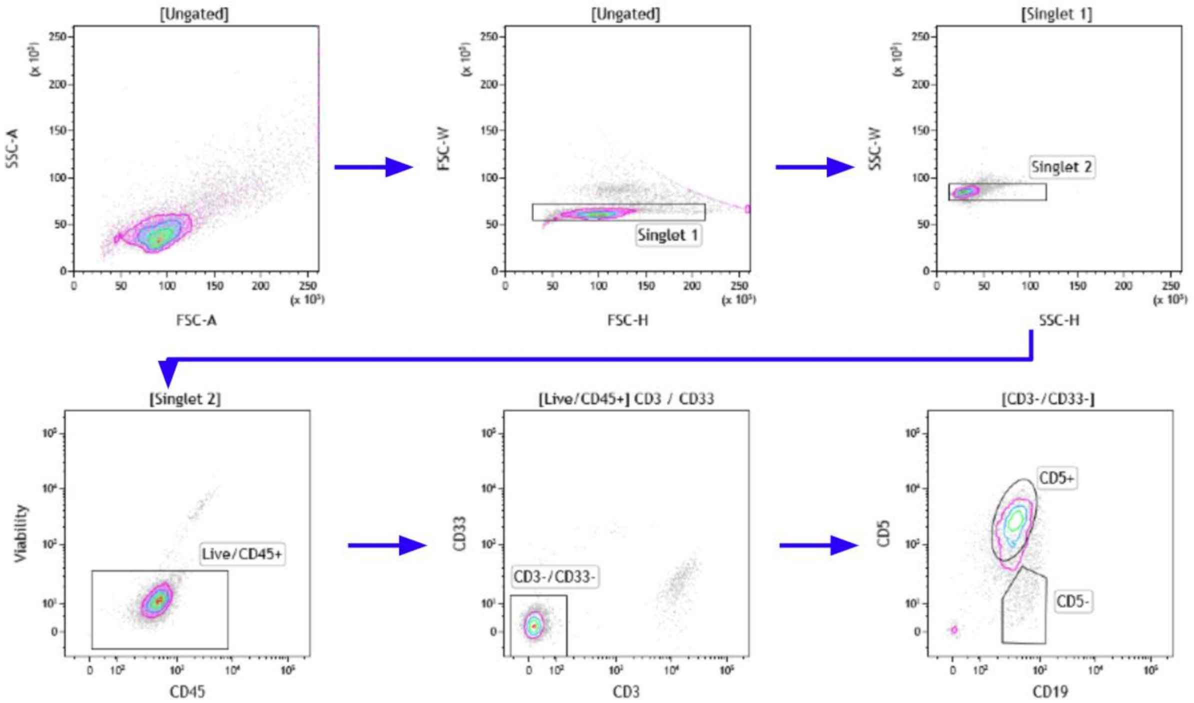

Figure S1. FACS parameter diagrams for the enrichment of CD5+ and CD5- cells from a CLL patient PBMC sample. FACS flow diagrams depicting the steps involved in the enrichment of CD5+ cells and CD5- cells from live CD45+, CD3-, CD33-, CD19+ cells, followed by separation into either CD5+ or CD5- collection tubes (Figure 1). FACS: Fluorescence Activated Cell Sorting.

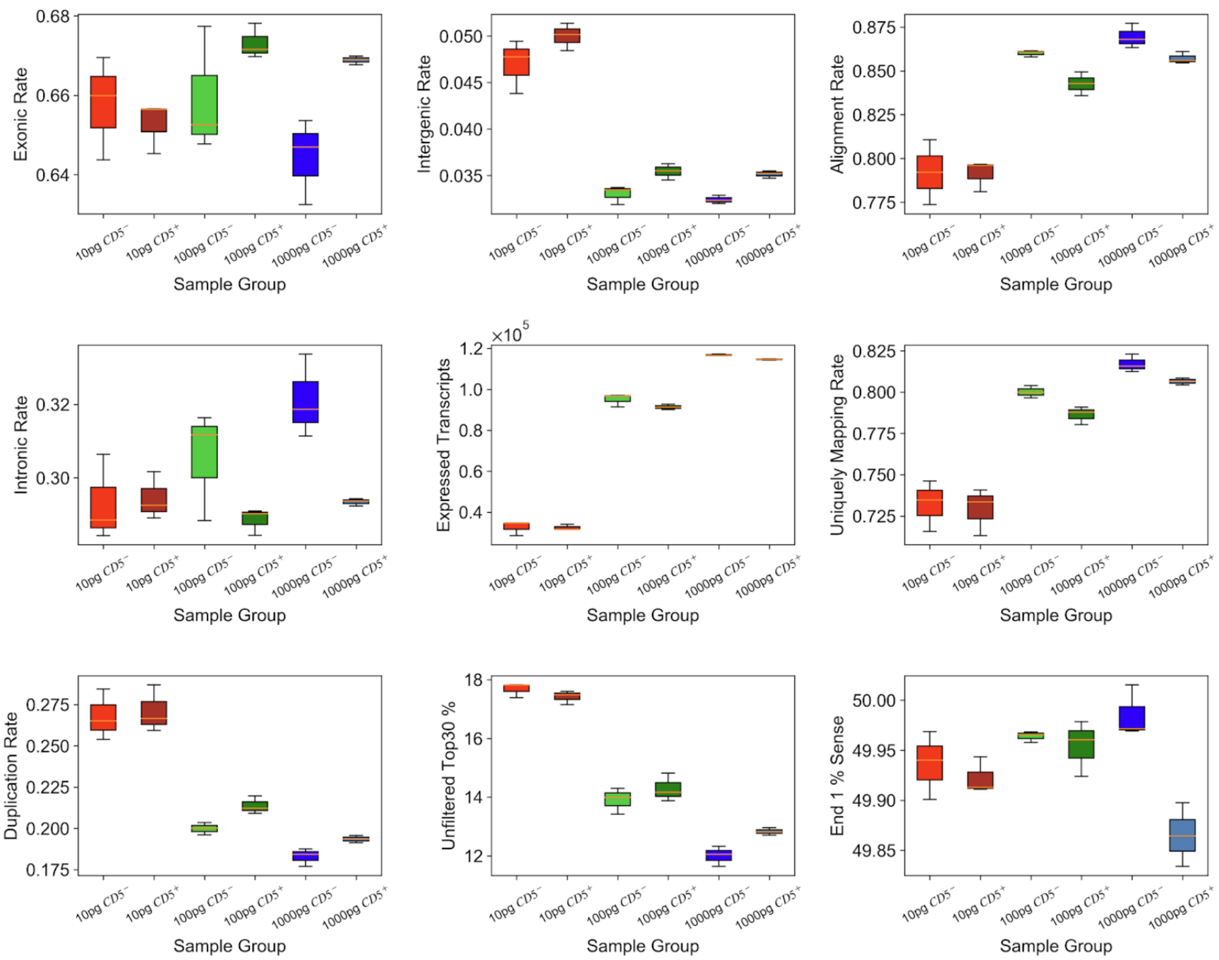

Figure S2. Survey of quality control (QC) metrics of RNA-Seq data. All sequencing was subject to quality control as described in **Methods**. Key metrics are summarized here. Notably, in many metrics such as Intergenic Rate, Alignment Rate, and Duplication Rate, the 10-pg groups indicate lower quality libraries than 100-pg and 1000-pg. “Top30” corresponds to the proportion of reads that belong to the 30 highest genes by expression. Boxplots: orange line, mean metric value; whiskers: displaying 1.5X the inter-quartile range (IQR) beyond the first and the third quartiles; circles: outliers.

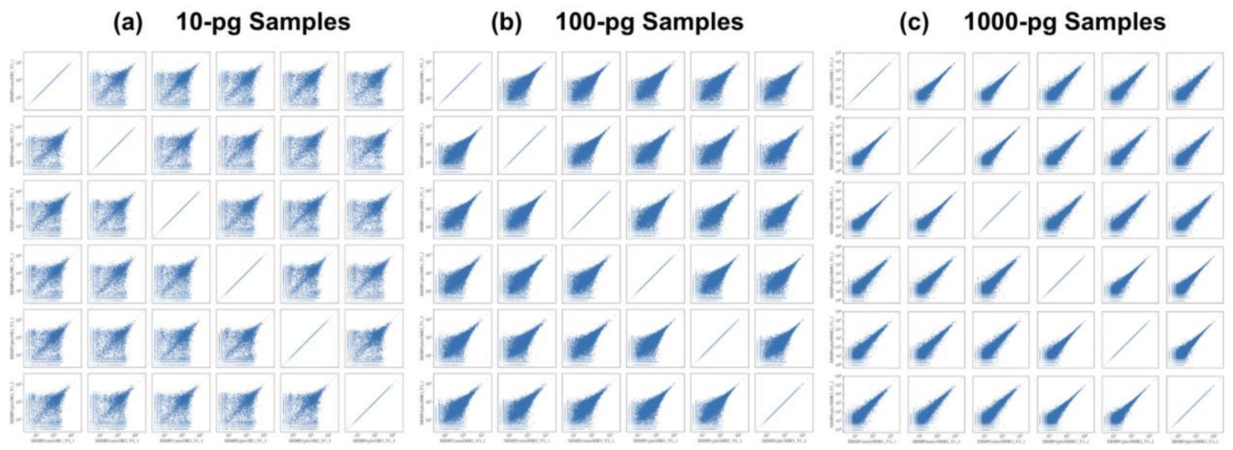

Figure S3. Between-sample correlations of detected RNA-Seq read counts. Scatter plots are drawn comparing each sample to each other sample for each input mass. 10-pg samples show much more scattered counts, whereas 100-pg and 1000-pg samples show progressively higher correlation.

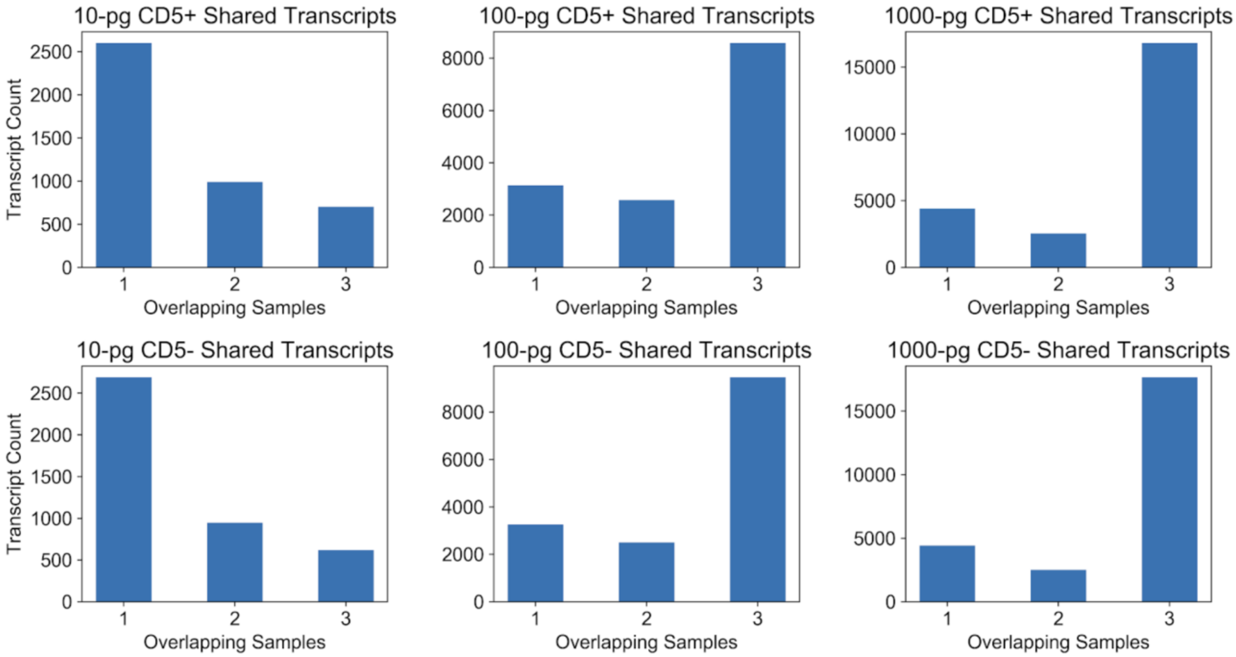

Figure S4. Comparison of overlapping transcripts. The analysis from Figure 3a was repeated, although CD5- and CD5+ samples were considered separately. Notably, the trend between CD5+ and CD5- mirrors that of the pooled data in Figure 3a.

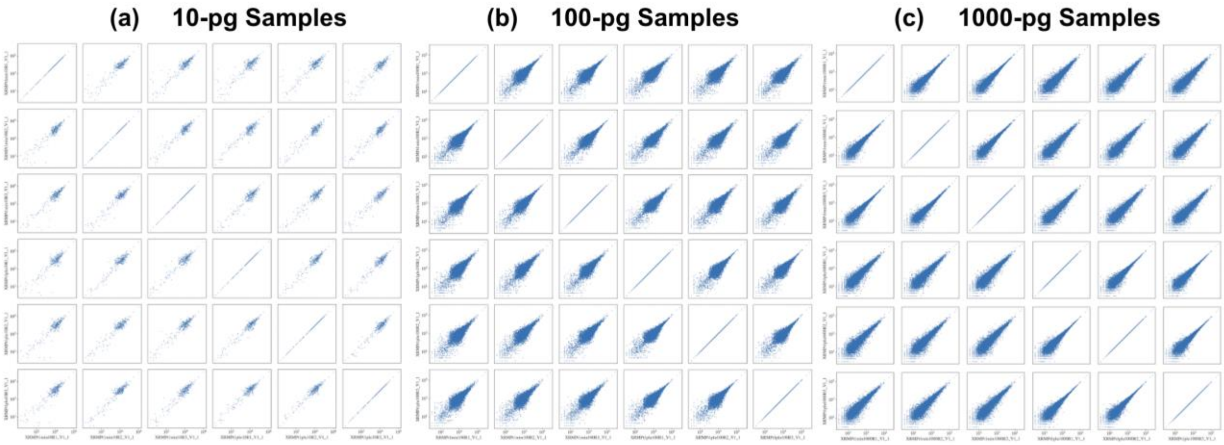

Figure S5. CLEAR Filtering results in fewer noisy transcripts at the 10-pg sample level. Analysis from Figure S3 was repeated using CLEAR-filtered gene counts. Notably, 10-pg samples are observed to be sparser, while the remaining data points are of much higher correlation.

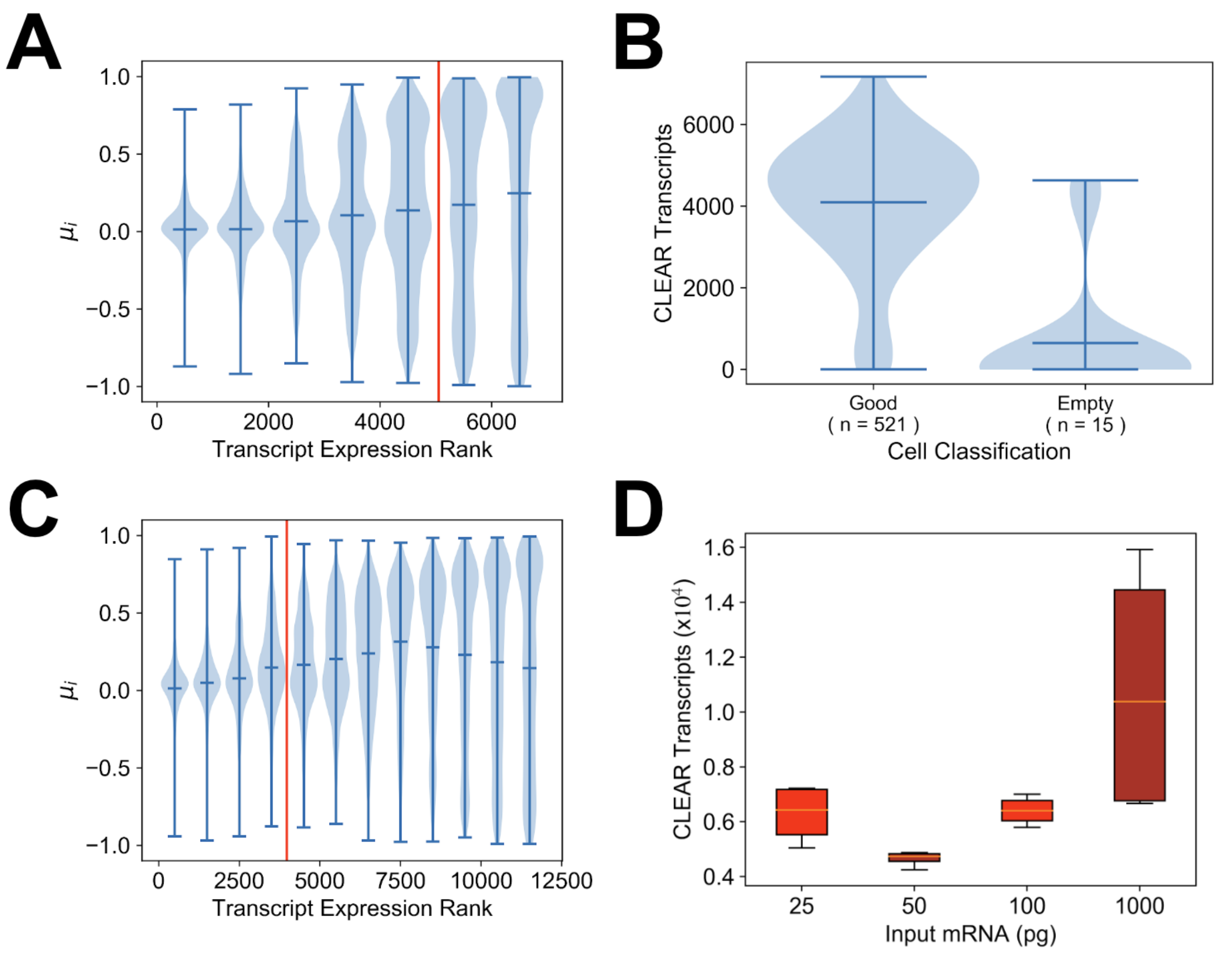

Figure S6. Application of CLEAR to public datasets. A-B) Data from Ilicic et al. [25] was processed using the CLEAR pipeline; **C-D)** Data from Bhargava et al. [14] was processed using the CLEAR pipeline; **A)** An example CLEAR trace from released data shows a representative separation; **B)** CLEAR transcript identity allows the separation of cells the authors classified as "Empty" from those classified as "Good." **C)** An additional example trace; **D)** CLEAR transcript counts are indicative of the input mRNA mass used to generate a sequencing library.

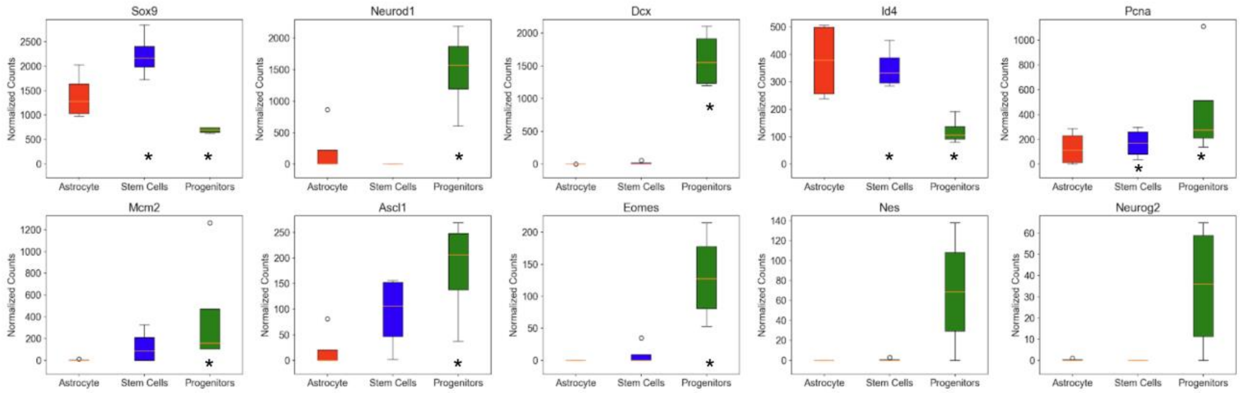

Figure S7. Neuronal cell type markers which did not pass the CLEAR criterion. Similar to Figure 4d, for each remaining gene, expression was plotted using the raw counts. Individual cell types which passed CLEAR filtering are indicated with an asterisk (*) below the respective box plot. Boxplots: orange line, mean CLEAR transcripts for four biological replicates per neural cell type; whiskers: displaying 1.5X the inter-quartile range (IQR) beyond the first and the third quartiles; circles: outliers.
